# To Defer or Not Defer? The Challenges of Physiology in Acute Coronary Syndromes

**DOI:** 10.5935/abc.20180206

**Published:** 2018-10

**Authors:** Carlos M. Campos, Pedro A. Lemos

**Affiliations:** 1 Hospital Israelita Albert Einstein, São Paulo, SP - Brazil; 2 Instituto do Coração (InCor), Faculdade de Medicina da Universidade de São Paulo, São Paulo, SP - Brazil

**Keywords:** Acute Coronary Syndrome/physiopathology, Percutaneous Coronary Intervention, Fractional Flow Reserve, Myocardial, Angina, Stable, Myocardial Revascularization

Conventional angiography may unreliably estimate the functional severity of coronary lesions, particularly of intermediate stenosis.^1^ It is in this context that intracoronary physiology, namely the measurement of fractional flow reserve (FFR), has been developed: to precisely differentiate stenoses that cause myocardial ischemia from those that are not significantly obstructive. Overall, FFR has been applied as a decision-making tool, helping to indicate (or defer) revascularization in intermediate or ambiguous coronary stenoses.^2^ Compared with angiography alone, the addition of FFR-derived information has been shown to improve patient outcomes and procedural cost-efficiencies, with physiology-guided coronary revascularization being currently recommended in clinical practice guidelines, on the grounds of ample scientific evidence.^3^

Almost twenty years ago, the pivotal DEFER trial consolidated the concept that FFR-based postponement of revascularization is safe.^4^ However, numerous reasons make the translation of the DEFER trial to contemporary clinical practice outdated: i) the excessively restrictive 0.75 cutoff (as used in the study) has been supplanted by the more permissive 0.80 threshold, ii) balloon angioplasty as a stand-alone therapy has been largely replaced by drug-eluting stents, iii) more potent antiplatelet agents and other medical therapies have become available, and iv) the relation between FFR and the obstructive profile of coronary lesion is yet being questioned by some authors.^5^ Thus, the contemporary safety of deferring lesions in stable angina pectoris (SAP) and acute coronary syndrome (ACS) on the basis of FFR still deserves investigation.

In this issue of Arquivos Brasileiros de Cardiologia, Martins et al.^6^ investigated the relative risks of deferring lesions in patients with SAP and ACS. The authors used a metanalysis of 1 prospective and 6 observational studies to compare the rates of events between these 2 groups of clinical presentations (n = 5107). There was no difference for all-cause mortality (relative risk (RR) = 1.44; (95% CI, 0.9-2.4), cardiovascular mortality (RR = 1.29, 95% CI = 0.4-4.3) and target vessel revascularization (RR = 1.46, 95% CI = 0.9-2, 3) for FFR-based revascularization within patients with ACS and SAP. However, there was a higher risk of myocardial infarction (RR = 1.83, 95% CI = 1.4-2.4) in deferring lesions without functional significance in patients with ACS.

By definition, any metanalysis serves from the amalgam of data comprised by works previously performed. Metanalyses, therefore, may become outdated, and need to be re-processed as fresh data is released in the literature. Recently, Escaned et al.^7^ assessed the safety of the deferral of coronary revascularization based on invasive functional evaluation (instantaneous wave-free ratio [iFR] and FFR.^7^ The safety of deferral of coronary revascularization in the pooled per-protocol population (n = 4,486) of the DEFINE-FLAIR (Functional Lesion Assessment of Intermediate Stenosis to Guide Revascularisation) and iFR-SWEDEHEART (Instantaneous Wave-Free Ratio Versus Fractional Flow Reserve in Patients With Stable Angina Pectoris or Acute Coronary Syndrome) randomized clinical trials was investigated. Unfortunately, this study was not included in the metanalysis by Martins et al.^6^. Escaned et al.^7^ demonstrated that, overall, deferral of revascularization is equally safe with both iFR and FFR, with a low MACE rate of about 4%. The clinical presentation with ACS was associated with a higher MACE (MACE = major adverse cardiac events, defined as the composite of all-cause death, nonfatal myocardial infarction, or unplanned revascularization at 1 year) rate compared with SAP in deferred patients (5.91% vs. 3.64% in ACS and SAP, respectively; fully adjusted hazard ratio: 0.61 in favor of SAP; 95% confidence interval: 0.38 to 0.99; p = 0.04).

The higher risk for physiology-based stenosis deferral in patients with ACS may reflect the different physiological conditions from those with SAP. The microcirculatory vasodilation during hyperemia may be transiently affected in the acute phase of ACS, also in territories far from the culprit lesions.^8^ Another factor related to this higher prevalence of events in ACS may be the widespread coronary inflammation in these patients.^8^ Buffon et al.^9^ have shown a depletion of the neutrophil myeloperoxidase content in blood from the great cardiac and femoral vein in patients with ACS, regardless of the site of the stenosis.^9^ This was not present in patients with stable angina and multiple stenosis, patients with variant angina and recurrent ischemia, or controls. The myeloperoxidase content is an index of advanced inflammatory activation and its depletion in ACS can be translated as a widespread activation of neutrophils across the coronary vascular bed.

Today, interventional cardiologists have a vast diagnostic armamentarium to be used in the cath lab as adjunctive tools (e.g. FFR, intravascular ultrasound, optical coherence tomography). The question to be answered in the coming years is how to align the currently available scientific information to provide the best decision algorithms in selecting the most appropriate candidates for myocardial revascularization.
